# Increased circular RNA UBAP2 acts as a sponge of miR-143 to promote osteosarcoma progression

**DOI:** 10.18632/oncotarget.18671

**Published:** 2017-06-27

**Authors:** Hao Zhang, Guangchao Wang, Chen Ding, Peng Liu, Renkai Wang, Wenbin Ding, Dake Tong, Dajiang Wu, Cheng Li, Qiang Wei, Xin Zhang, Di Li, Peizhao Liu, Haochen Cui, Hao Tang, Fang Ji

**Affiliations:** ^1^ Department of Orthopedics, Changhai Hospital, Second Military Medical University, Shanghai, China; ^2^ Department of General Sugery, Changhai Hospital, Second Military Medical University, Shanghai, China

**Keywords:** circRNA, circUBAP2, osteosarcoma, miR-143, Bcl-2

## Abstract

Deregulated expression of circular RNA (circRNA) has been determined to be important in carcinogenesis and progression; however, in the most common type of primary malignant bone tumor osteosarcoma, the roles of circRNA in cancer development still remain to be elucidated. Here, we found that circRNA UBAP2 (circUBAP2) expression is significantly increased in human osteosarcoma tissues as compared to those in matched controls. Increased circUBAP2 expression was significantly correlated with human osteosarcoma progression and prognosis. Furthermore, increased circUBAP2 could promote osteosarcoma growth and inhibit apoptosis both *in vitro* and *in vivo*. Mechanistically, circUBAP2 was found to inhibit the expression of microRNA-143 (miR-143), thus enhancing the expression and function of anti-apoptotic Bcl-2, which is a direct target of miR-143. Together, our results suggest the roles of circUBAP2 in osteosarcoma development and implicate its potential in prognosis prediction and cancer therapy.

## INTRODUCTION

Circular RNA (circRNA) is a new class of endogenously expressed non-coding RNA, which is characterized by covalently closed loop structures with neither 5’ to 3’ polarity nor polyadenylated tail [1, 2]. CircRNA is conserved in mammals and seems to be specifically expressed in a cell type or developmental stage, which may indicate that it participates in diverse physiological and pathological processes [3–6]. Currently, it is accepted that endogenously expressed circRNAs contain conserved microRNA (miRNA) target sites, and function as miRNA sponges to regulate the expression of target genes in mammals. Up to now, the deregulation of circRNAs has been suggested in a set of human diseases, especially cancer progression [7–14]. However, it is still an ongoing process to reveal the expression profiles of circRNAs and elucidate and functions of deregulated circRNAs in cancer development.

Just like the extensive studies of microRNAs (miRNAs) in cancer biology, the roles of circRNAs in carcinogenesis and progression have attracted much attention. Recently, the expression profiles of circRNAs in some types of cancer tissues have been elucidated, such as colorectal cancer, hepatocellular carcinoma, and gastric cancer [15–18]. Among them, some deregulated circRNAs have been found to be potential biomarkers in cancer diagnosis and prognosis identification [19–23]. These observations have strongly suggested the potential usage of circRNAs in clinical practice for the treatment of cancer patients. However, it remains to be investigated that whether circRNAs are deregulated and participate in the carcinogenesis and progression of osteosarcoma.

Osteosarcoma is the third most common cancer in childhood and young adults and the most common cancer of bone characterized by an aggressive clinical course [24]. For patients having no metastatic disease at diagnosis, the 5-year survival is 60% to 70% [25]. However, for patients who present with metastatic disease or whose tumor recurs, the clinical outcomes are far worse [26]. Currently, the mechanisms responsible for the oncogenic insults in the initiation and progression of osteosarcoma are still not fully elucidated. To date, non-coding RNAs including miRNAs and long non-coding RNAs have been extensively studied in osteosarcoma development [27–29]. However, as a new class of endogenously expressed non-coding RNA, the roles and mechanisms of circRNAs in osteosarcoma carcinogenesis and progression still require further investigation.

In order to identify the deregulated circRNAs in osteosarcoma, we obtained paired clinically resected osteosarcoma tissues and adjacent normal tissues, and examined the expression profile of circRNAs using microArray in this study. CircRNA UBAP2 (circUBAP2) was found to be the most markedly increased circRNA in osteosarcoma tissue as compared to that in matched adjacent control. Thus, we focused on the roles and underlying mechanisms of circUBAP2 in osteosarcoma development, including clinical stages, patient survival, and cancer biology properties, so as to suggest new mechanisms of osteosarcoma progression and potential targets for cancer treatment and prognosis prediction.

## RESULTS

### The expression of circRNA circUBAP2 was increased in osteosarcoma

**Figure 1:**
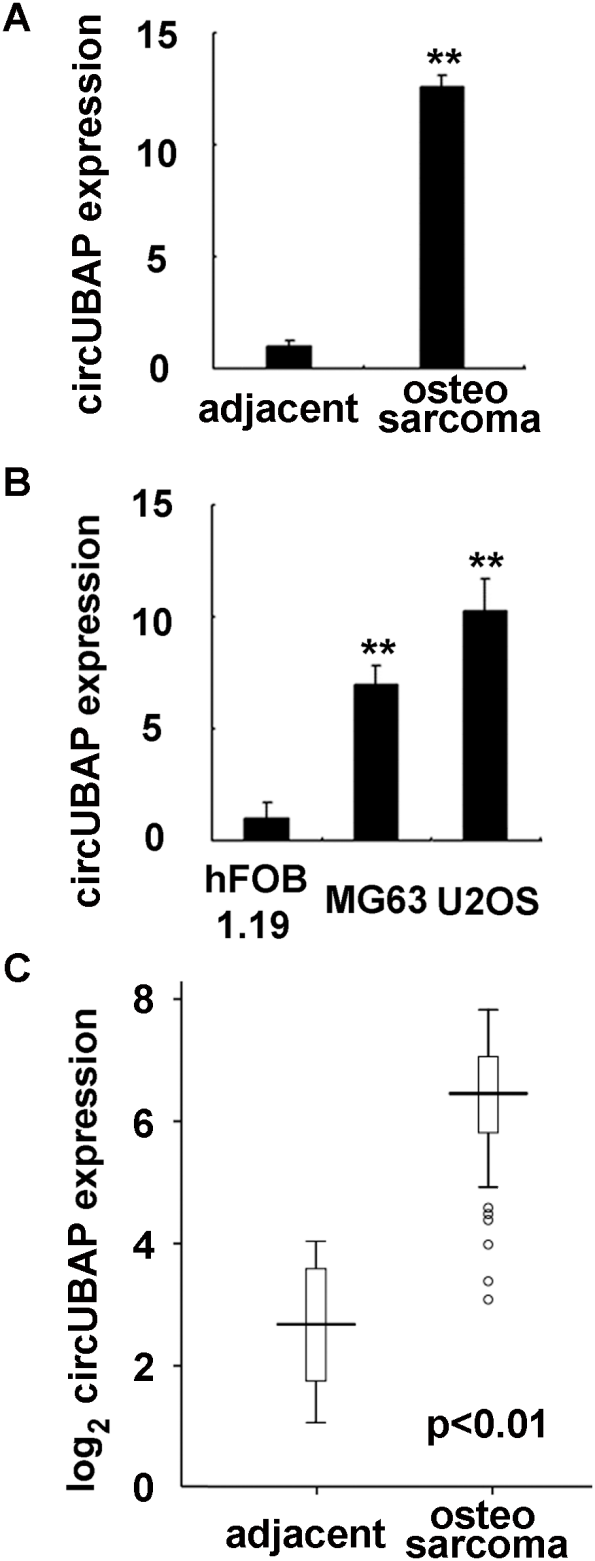
CircUBAP2 expression is increased in osteosarcoma tissues and cell lines **(A)** circUBAP2 expression in osteosarcoma tissues and matched adjacent nontumor tissues was shown as indicated. **(B)** circUBAP2 expression was examined in human osteoblast cell line hFOB 1.19 and osteosarcoma cell lines MG63 and U2OS using qRT-PCR. **(C)** In 92 paired human osteosarcoma tissues and matched adjacent normal tissues, circUBAP2 expression was examined using qRT-PCR. Data are shown as mean ± s.d. (n = 3) (A, B), or as the horizontal lines (median), the boxes (interquartile range), and the whiskers (2.5th and 97.5th percentiles) in C. **, *p<0.01*.

In order to investigate the expression profile of circRNAs in osteosarcoma, we used human osteosarcoma tissue and matched adjacent normal tissue, and applied circRNA microArray analysis form Arraystar to examine the deregulated circRNAs in osteosarcoma. As shown in Figure 1A, circRNA circUBAP2 was found to be the most markedly increased circRNA in osteosarcoma tissue as compared to that in matched normal tissue. Hence, we intend to focus on the roles and underlying mechanisms of circUBAP2 in human osteosarcoma development. CircUBAP2 expression in osteosarcoma was further determined in osteosarcoma cell lines, and we found that circUBAP2 expression was also significantly increased in human osteosarcoma cell lines MG63 and U2OS as compared to that in normal osteoblastic cell line hFOB 1.19 (Figure 1B). Furthermore, in the 92 pairs of human osteosarcoma and adjacent normal tissue specimens, circUBAP2 expression was determined to be significantly increased in osteosarcoma tissues (Figure 1C). Thus, these data determine that circUBAP2 expression is significantly increased in osteosarcoma.

### Increased circUBAP2 expression is significantly correlated with the progression and prognosis of osteosarcoma patients

**Figure 2:**
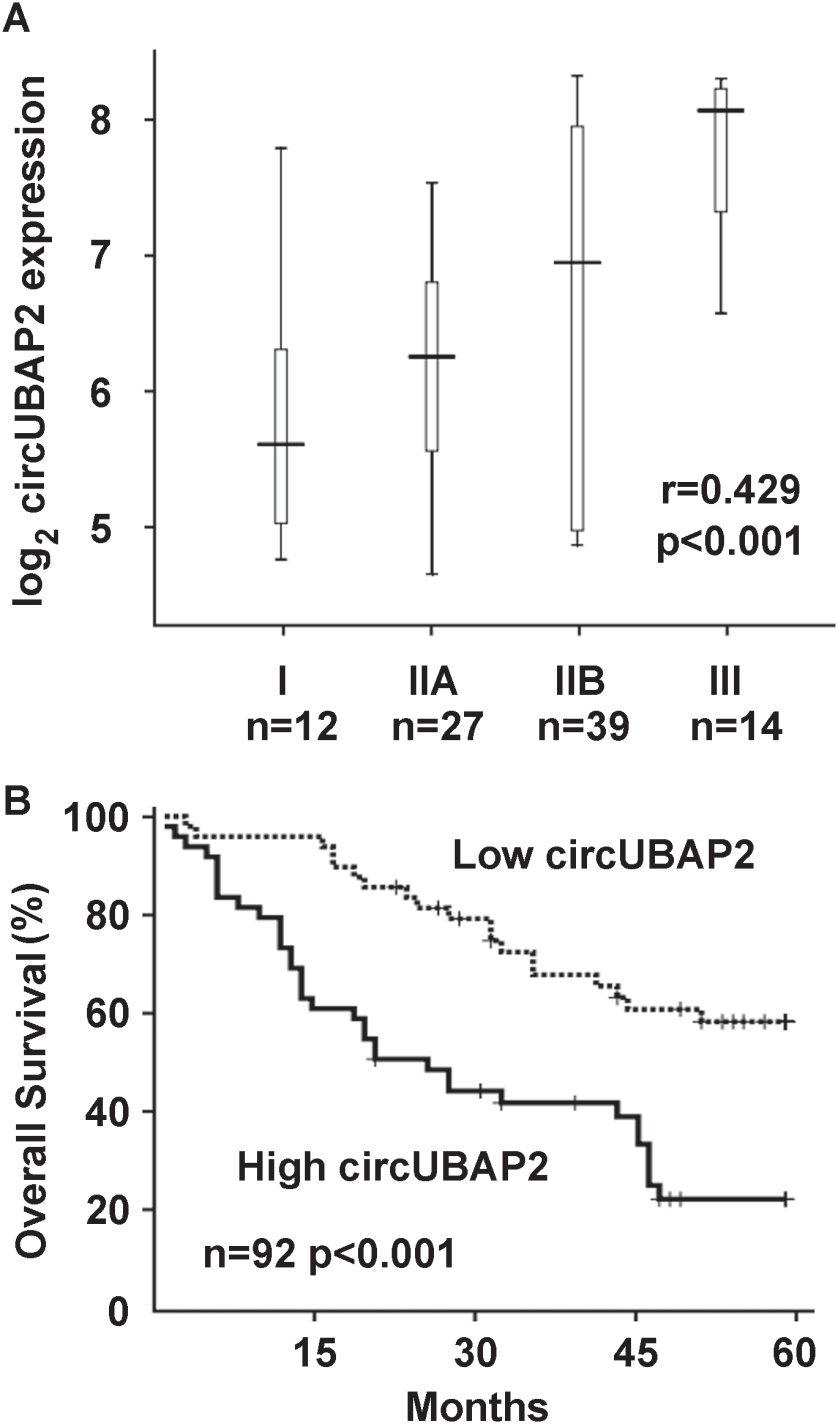
Increased circUBAP2 expression is correlated with osteosarcoma progression and prognosis **(A)** Correlation between circUBAP2 and osteosarcoma stages from I to III were analyzed by Spearman's rank correlation assay with the *r* and *p* values indicated. **(B)** Kaplan-Meier survival analysis in the 92 osteosarcoma patients according to circUBAP2 expression in tumor. The median value of circUBAP2 expression in tumor was chosen as the cut-off.

As circUBAP2 was found to be significantly increased in osteosarcoma tissues, we then examined whether increased circUBAP2 expression was correlated with osteosarcoma progression and patient survival. In the examined 92 human osteosarcoma tissue specimens, circUBAP2 expression was found to be significantly positive-correlated with tumor stages of osteosarcoma using Spearman's rank correlation assay (Figure 2A). Moreover, increased circUBAP2 expression in osteosarcoma tissues was significantly correlated with reduced survival and poor prognosis, shown as the Kaplan-Meier survival analysis of overall survival in these patients (Figure 2B). Taken together, these results suggest that increased circUBAP2 expression may be important in osteosarcoma progression and prognosis, and examination of circUBAP2 expression may be useful in pathological identification and prognosis prediction of osteosarcoma.

### CircUBAP2 promotes osteosarcoma growth both *in vitro* and *in vivo*

**Figure 3:**
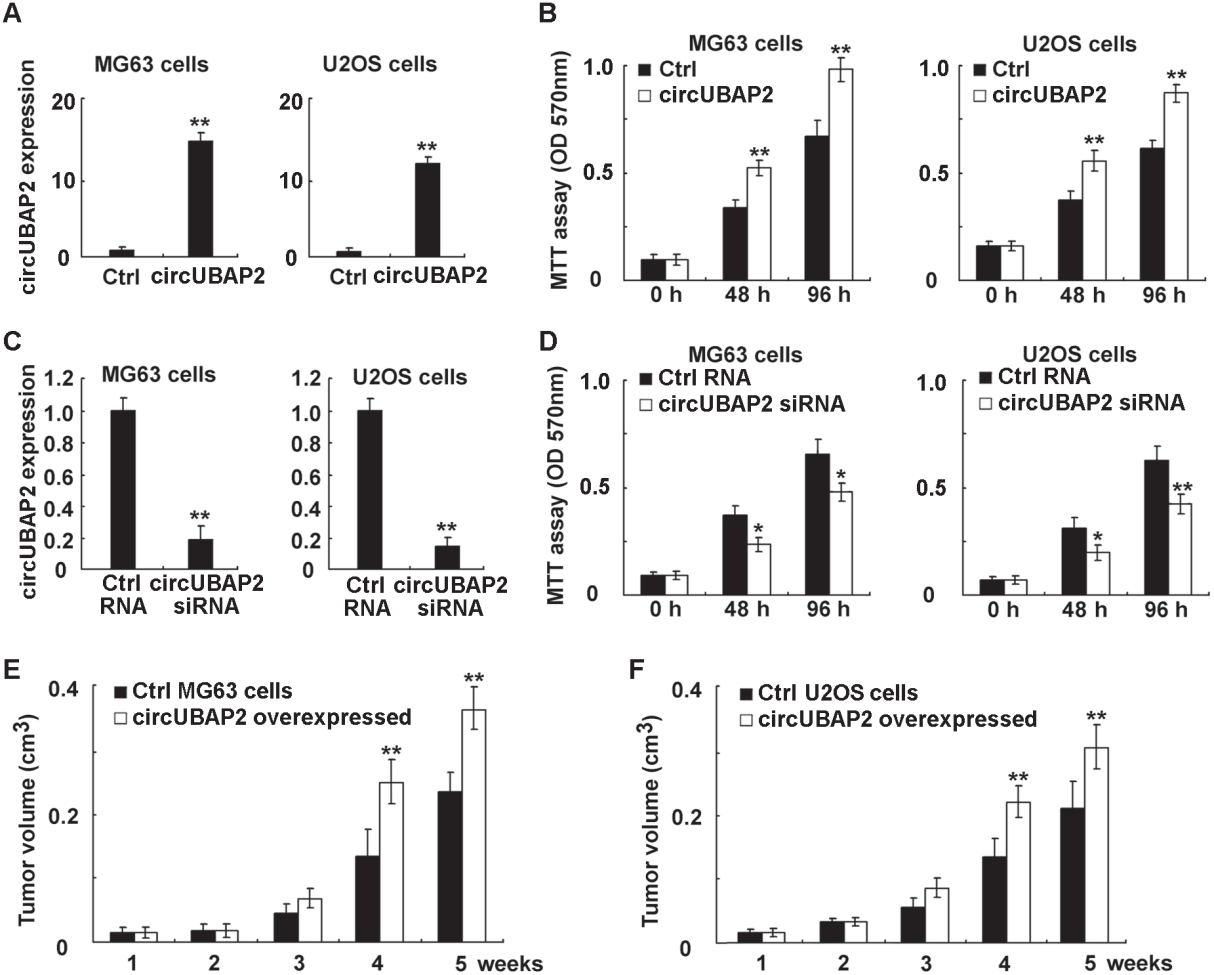
CircUBAP2 promotes osteosarcoma growth both *in vitro* and *in vivo* **(A, B)** Osteosarcoma MG63 and U2OS cells were transfected with circUBAP2 expressing plasmids, and circUBAP2 expression was examined using qRT-PCR and cell proliferation was measured using MTT assay. **(C, D)** Osteosarcoma MG63 or U2OS cells were transfected with circUBAP2 siRNA, and circUBAP2 expression was examined using qRT-PCR and cell proliferation was measured using MTT assay. **(E, F)** Ctrl osteosarcoma cell lines or circUBAP2 stably overexpressed osteosarcoma cells were injected subcutaneously into the nude mice. Tumor growth was examined and the curves were shown as indicated. Data are shown as mean ± s.d. (n = 4). Similar results were obtained in three independent experiments. *, *p*<0.05; **, *p*<0.01.

Because circUBAP2 was increased in osteosarcoma and corrected with poor prognosis, we next examined whether circUBAP2 functioned as an oncogene in osteosarcoma. In osteosarcoma MG63 and U2OS cells, we found that circUBAP2 overexpression could promote cell proliferation in both cell lines (Figure 3A and 3B). Accordingly, knockdown of circUBAP2 expression suppressed cell proliferation in osteosarcoma cells (Figure 3C and 3D). Furthermore, in circUBAP2 stably overexpressed osteosarcoma cell lines, cell growth *in vivo* was significantly promoted as compared to that in control cells (Figure 3E and 3F). Hence, these results show that circUBAP2 may be an oncogene to promote osteosarcoma growth, and inhibition of circUBAP2 expression may have therapeutic potential in osteosarcoma.

### CircUBAP2 inhibits osteosarcoma cell apoptosis by upregulating the expression of anti-apoptotic Bcl-2

**Figure 4:**
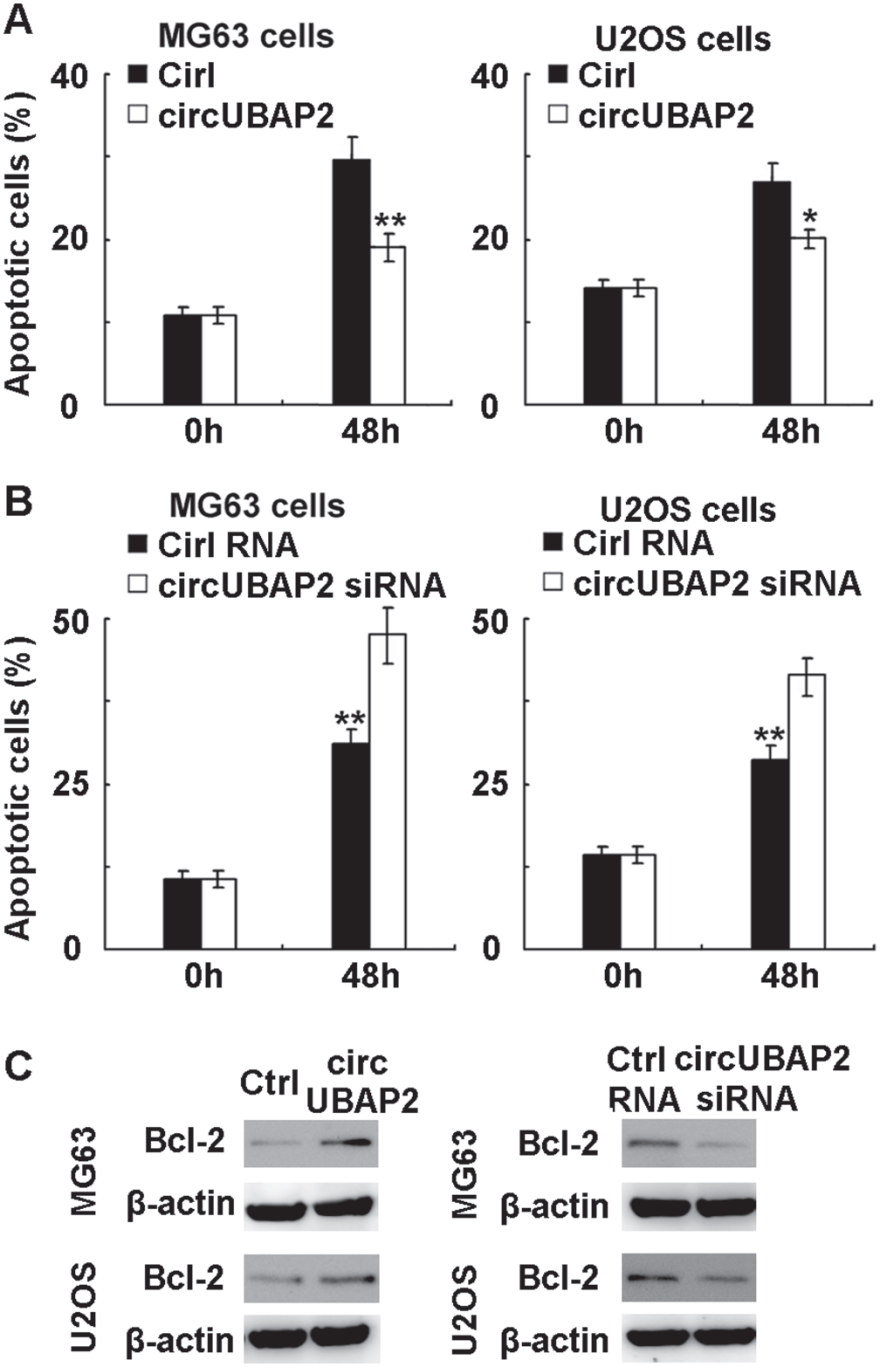
CircUBAP2 inhibits apoptosis by upregulating anti-apoptotic Bcl-2 expression **(A)** MG-63 and U2OS cells were transfected with circUBAP2 expressing plasmids, and then subjected to serum deprivation and hypoxia. Cell apoptosis was detected by Annexin V-PI staining. **(B)** MG-63 and U2OS cells were transfected with circUBAP2 siRNA, and then subjected to serum deprivation and hypoxia. Cell apoptosis was detected by Annexin V-PI staining. **(C)** MG63 and U2OS cells were transfected as in A and B, Bcl-2 expression was detected using Western blot. Data are shown as mean ± s.d. (n = 4) or as one representative experiment. Similar results were obtained in three independent experiments. *, *p*<0.05; **, *p*<0.01.

The mechanism responsible for the growth promoting effect of circUBAP2 on osteosarcoma cells was next investigated. We examined the apoptosis and cell cycle progression in osteosarcoma cells by circUBAP2 overexpression, and found that circUBAP2 overexpression significantly inhibited cell apoptosis upon serum deprivation and hypoxia in osteosarcoma MG63 and U2OS cells (Figure 4A). Moreover, knockdown of circUBAP2 expression significantly promoted cell apoptosis of osteosarcoma cells (Figure 4B). Thus, we conclude that circUBAP2 promotes osteosarcoma growth by inhibiting cell apoptosis.

The underlying mechanism responsible for the inhibitory effect on cell apoptosis mediated by circUBAP2 was then studied. We screened the expression of intracellular molecules associated with cell apoptosis in circUBAP2 overexpressed MG63 and U2OS cells, including Bcl-2, Mcl-1, Bax, Bim, Bad, Ras, Bid, FADD, Bcl-xL, PTEN, XIAP, and phosphorylated ERK and Akt, and found that anti-apoptotic Bcl-2 expression was significantly increased by circUBAP2 overexpression (Figure 4C). Furthermore, knockdown of circUBAP2 inhibited the expression of Bcl-2 in MG63 and U2OS cells. As Bcl-2 is an important anti-apoptotic molecule well-accepted to protect osteosarcoma cells from apoptosis [30, 31], we conclude that circUBAP2 may inhibit osteosarcoma cell apoptosis by upregulating Bcl-2 expression.

### CircUBAP2 binds miR-143 in osteosarcoma cells

**Figure 5:**
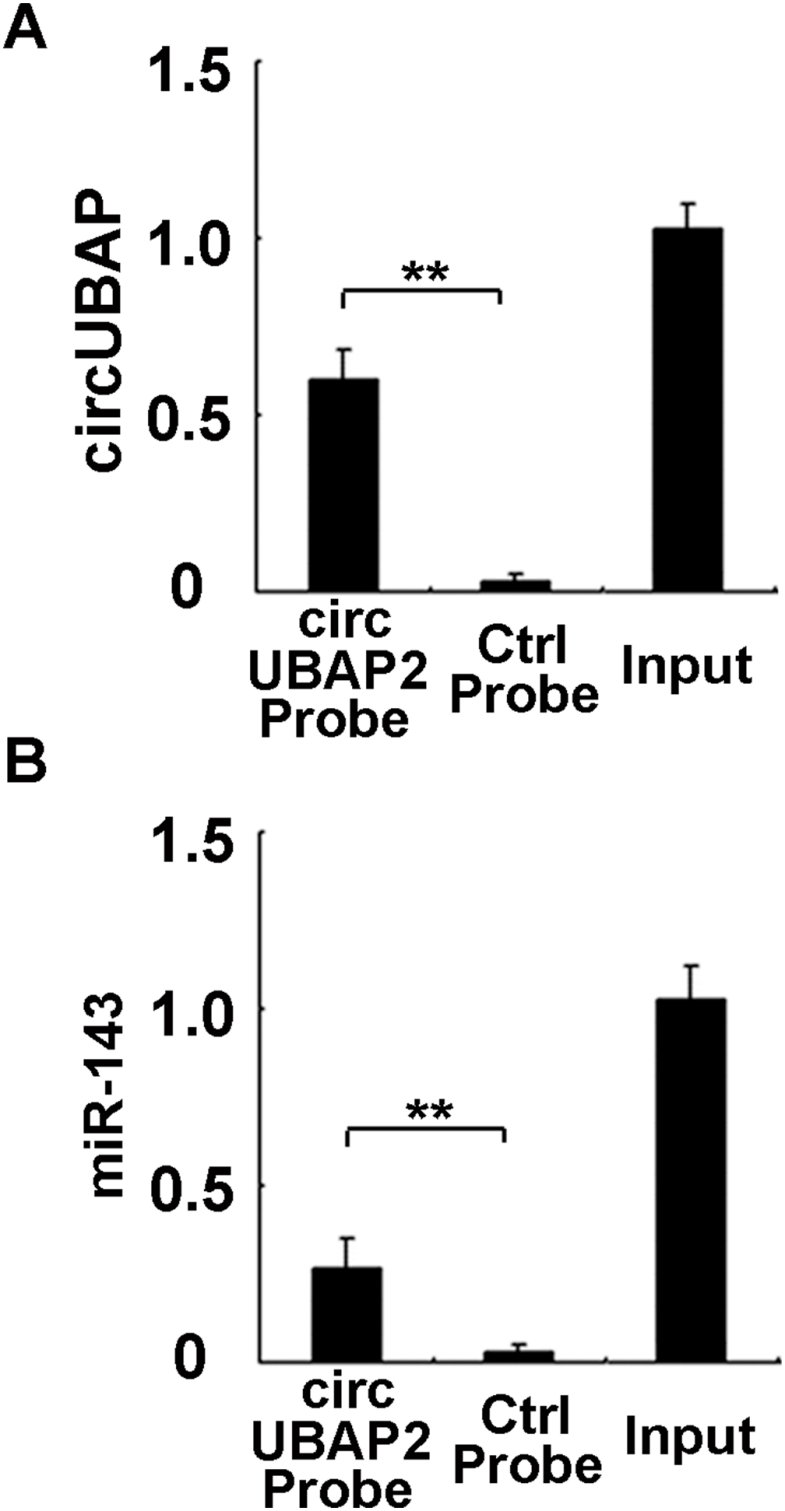
CircUBAP2 binds miR-143 in osteosarcoma cells In circUBAP2 stably overexpressed MG63 cells, circUBAP2 was precipitated using circUBAP2 specific probe or control probe, circUBAP2 **(A)** and miR-143 **(B)** were detected in the precipitates using qRT-PCR. Data are shown as mean ± s.d. (n = 4). Similar results were obtained in three independent experiments. **, *p*<0.01.

CircRNAs may function as miRNA sponge to bind miRNAs and regulate expression of target genes. We then analyzed the association between circUBAP2 and miRNAs. Using MiRanda prediction algorithms (http://www.microrna.org/), a set of miRNAs were predicted to have potential interaction with circUBAP2, including miR-150, miR-135, miR-101, miR-181, miR-23, miR-149, miR-139, miR-491, miR-124, miR-301m miR-328, miR-122, miR-186, let-7, miR-132, miR-191, miR-425, miR-125, miR-149, miR-143, and miR-146a. To find the direct interacted miRNAs with circUBAP2 in osteosarcoma cells, circUBAP2 specific probe was used to perform RNA precipitation (RIP) as reported [32]. CircUBAP2-associated RNAs were precipitated by the specific probe, and the potential miRNAs predicted by miRanda were detected using qRT-PCR. Among them, we found a specific enrichment of miR-143 in the precipitates of circUBAP2 (Figure 5A and 5B), suggesting that miR-143 is the circUBAP2-associated miRNA in osteosarcoma cells.

### CircUBAP2 functions as the sponge of miR-143 to inhibit apoptosis by upregulating anti-apoptotic Bcl-2 in osteosarcoma

**Figure 6:**
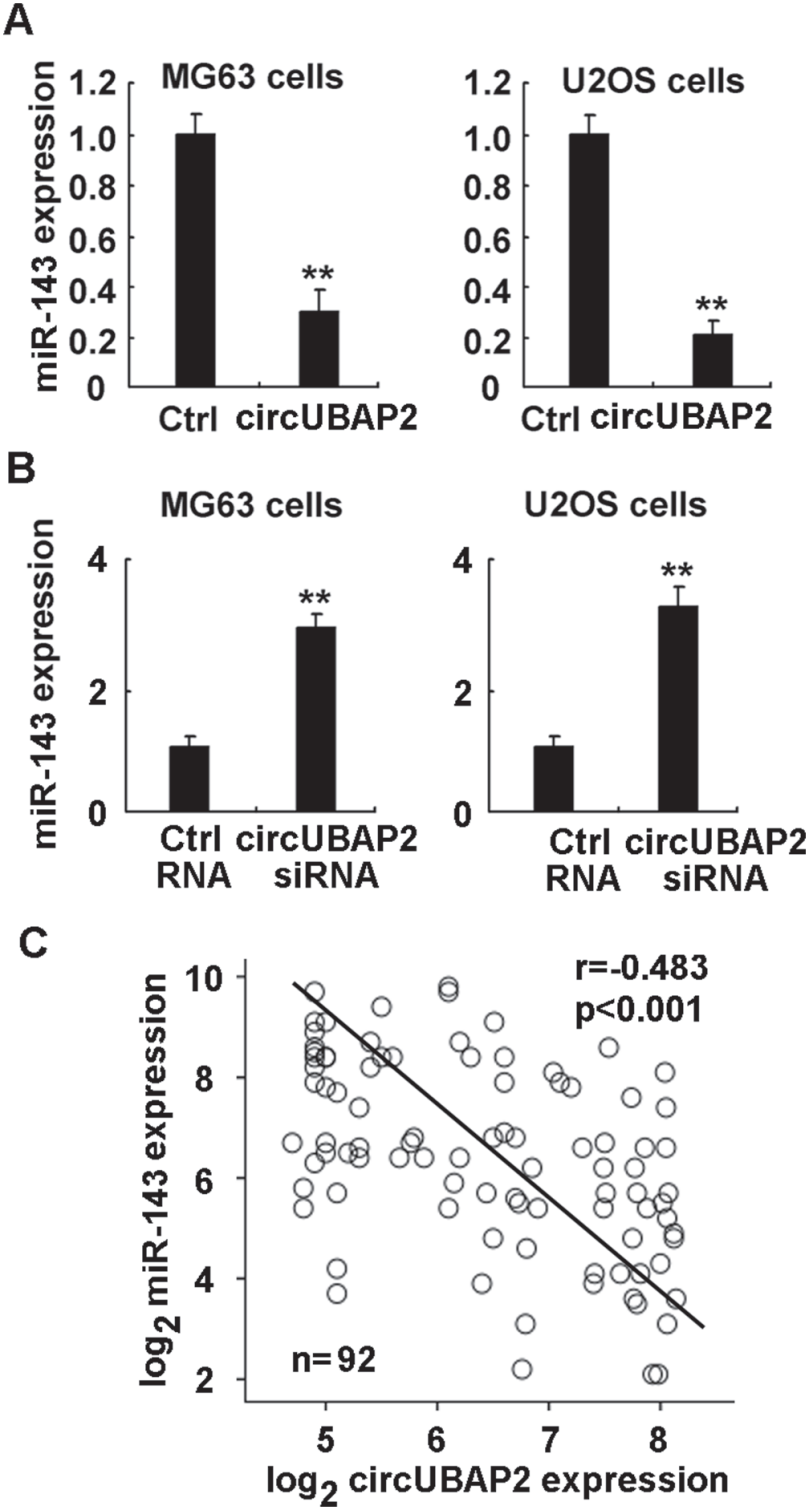
CircUBAP2 functions as the sponge of miR-143 **(A, B)** MG63 and U2OS cells were transfected with circUBAP2 expressing plasmids (A) or siRNA (B) as indicated, miR-143 expression was examined using qRT-PCR. **(C)** Correlation between miR-143 and circUBAP2 level in osteosarcoma tissues was statistically analyzed by Pearson’s correlation coefficient assay, and *r* and *p* values were shown as indicated. Data are shown as mean ± s.d. (n = 4). Similar results were obtained in three independent experiments. **, *p*<0.01.

In osteosarcoma, we previously reported that anti-apoptotic Bcl-2 was directly targeted by miR-143, and miR-143 is downregulated in osteosarcoma and causes the upregulation of anti-apoptotic Bcl-2 [33]. As circUBAP2 was found to bind miR-143, we next examined that whether circUBAP2 could function as the sponge of miR-143 and inhibit miR-143 expression, thus upregulating Bcl-2 expression in osteosarcoma. In MG63 and U2OS cells, overexpression of circUBAP2 inhibited the expression of miR-143, while knockdown of circUBAP2 enhanced miR-143 expression (Figure 6A and 6B). Furthermore, in human osteosarcoma tissues, circUBAP2 expression was found to be reverse-correlated with miR-143 expression, confirming its sponge function to inhibit miR-143 expression (Figure 6C). Thus, we conclude that circUBAP2 is the sponge of miR-143 and then upregulates anti-apoptotic Bcl-2 expression in osteosarcoma.

## DISCUSSION

Osteosarcoma is characterized by an aggressive clinical course and is the most common primary malignant bone tumor. Recently, molecular mechanisms responsible for osteosarcoma carcinogenesis and progression have attracted much attention in investigating osteosarcoma development. A set of deregulated non-coding RNAs have been identified to be important in osteosarcoma pathogenesis, including miRNAs and long non-coding RNAs [34]. In this study, we found that circUBAP2 expression is significantly upregulated in osteosarcoma, and circUBAP2 could promote osteosarcoma growth by enhancing anti-apoptotic Bcl-2 expression. Furthermore, increased circUBAP2 expression in human osteosarcoma tissues is suggested to be correlated with osteosarcoma progression and prognosis. Thus, we have presented that circUBAP2 may be a new prognosis predictor and therapeutic target in osteosarcoma.

Previously, we reported that miR-143 expression was decreased in osteosarcoma tissues, and miR-143 decrease was responsible for the increased anti-apoptotic Bcl-2 expression in osteosarcoma [33]. However, the underlying mechanism for miR-143 decrease in osteosarcoma was not known at that time. Here, we present that increased circUBAP2 expression is responsible for the decreased miR-143 expression in osteosarcoma, and circUBAP2 expression is reverse-correlated with miR-143 expression in human osteosarcoma tissues. Thus, the mechanism for miR-143 decrease in osteosarcoma may be dependent on the increased circUBAP2 expression. Moreover, the mechanism responsible for circUBAP2 upregulation in osteosarcoma is still unknown and requires further investigation.

Recently, it has attracted much attention to identify the molecular biomarkers which are correlated with cancer progression and prognosis of patients. We previously reported that a set of deregulated non-coding RNAs in osteosarcoma are correlated with cancer stages and prognosis of patients, including the decreased miR-133a and increased miR-148a [35–39]. Here, we presented that increased circUBAP2 expression in osteosarcoma is also corrected with cancer stages and prognosis of patients. As miRNAs and circRNAs are relatively more stable than other types of RNAs, combined detection of these deregulated miRNAs and circUBAP2 may be valuable to identify pathological stages and prognosis of osteosarcoma more accurately, and bears considerable potential in practice.

It is validated both *in vitro* and *in vivo* that circUBAP2 overexpression promotes osteosarcoma growth and progression. And inhibition of circUBAP2 can suppress osteosarcoma growth and induce apoptosis. Thus, it is suggested that circUBAP2 may be a potential therapeutic target of osteosarcoma. Moreover, as intervention approaches to inhibit gene expression *in vivo* are becoming reliable, especially the cholesterol-conjugated siRNAs for *in vivo* administration, we will further examine that whether inhibition of circUBAP2 expression *in vivo* can suppress osteosarcoma growth in our future study. The attempt of *in vivo* administration to inhibit circUBAP2 expression in osteosarcoma therapy may raise important and interesting future work in cancer treatment, especially for those who respond poorly to radiotherapy or chemotherapy.

## MATERIALS AND METHODS

### Patient samples

Human osteosarcoma tumor tissues and matched adjacent normal tissues were surgically resected from 92 primary osteosarcoma patients during operation. The detailed clinical information of these patients was presented previously [11, 12]. These tissues were quickly frozen in liquid nitrogen after surgical resection. All these samples were collected with the informed consents of these patients, and were approved by the ethics committee of Second Military Medical University, Shanghai, China.

### circRNA microArray

circRNA microArray of osteosarcoma tissue and matched control tissues were performed using circRNA chip provided by Arraystar containing the specific probes for human circRNA. Total RNA of tumor and control tissues was extracted using TRIZOL reagent (Invitrogen) and passed the quality control for RNA integrity. The samples were hybridized and washed in the circRNA microArray chips, and the signals were scanned and gathered as the standard protocol [35].

### qRT-PCR

Total RNA was extracted using TRIZOL reagent (Invitrogen) and reverse-transcribed using PrimeScript RT reagent Kit (Takara) following the standard protocol. qPCR was performed using SYBR Green I (Takara) and Lightcycler 2.0 (Roche). For evaluating the expression of circUBAP2, the primers were 5’-AGC CTC AGA AGC CAA CTC CTT TG-3’ (forward) and 5’-TCA GGT TGA GAT TTG AAG TCA AGA T-3’ (reverse). For miRNA analysis, stem-loop RT primer for miR-143 was 5'- GTC GTA TCC AGT GCA GGG TCC GAG GTA TTC GCA CTG GAT ACG ACG AGC TA -3', and q-PCR primers were 5'- AGT CAG TGA GAT GAA GCA CTG -3' (forward) and 5’- GTG CAG GGT CCG AGG T -3’ (reverse). The qRT-PCR analysis was performed and calculated as reported [40, 41].

### Cell culture and transfection

Human osteoblastic cell line hFOB 1.19, osteosarcoma cell lines MG63 and U2OS were cultured, seeded, and transfected as we described previously [33, 35, 36]. CircUBAP2 expressing plasmids were constructed into pcDNA3.1 vector (Invitrogen) and validated by sequencing. Plasmids were transfected using jetPrime transfection reagent (Polyplus-transfection) following the manufacturer’s instruction. CircUBAP2 specific siRNA and the control RNA were synthesized by GenePharma Company. siRNAs were transfected at the final concentration of 10 nM using INTERFERin transfection reagent (Polyplus-transfection) following the manufacturer’s instructions.

### Cell proliferation analysis

The *in vitro* cell proliferation assay of osteosarcoma MG63 and U2OS cells were presented as we reported previously [33, 35, 36]. In brief, cells were seeded into 96-well plates, transfected, and analyzed using MTT method in the indicated time periods. The absorbance was measured at 570 nm.

### Tumor growth analysis *in vivo*

All animal experiments were performed according to the National Institute of Health Guide for the Care and Use of Laboratory Animals. Osteosarcoma MG63 and U2OS cells (1×10^6^) were suspended in 0.1 ml PBS and injected subcutaneously into the posterior flank of BALB/c athymic nude mice. Tumor growth was measured as we described previously [33, 35, 36].

### Detection of apoptosis

Osteosarcoma MG63 or U2OS cells were transfected as indicated. At 48 hours post transfection, spent cell culture medium was replaced with serum free DMEM and subjected to hypoxia. In the indicated time periods post serum deprivation and hypoxia, cells were harvested, washed, resuspended in the staining buffer, and examined with Vybrant Apoptosis Assay kit (Invitrogen). Stained cells were detected by FACSCalibur and data were analyzed with CellQuest software (both from Becton Dickinson). The Annexin V-positive cells were regarded as apoptotic cells [33].

### circRNA precipitation

In CircUBAP2-overexpresed MG63 cells, the cells were fixed, lysed and sonicated. After centrifugation, 50 ul of the supernatant was used as the input control and the rest was incubated with circUBAP2 specific probes-streptavidin beads (Invitrogen). Biotin-labeled circUBAP2 probe was synthesized by Sangon Biotech, and the sequence is 5’-AAT TCT TTT TCT TAC ACC CTA CAG-3’. Then, the beads-probe-circRNA precipitates was washed and incubated with proteinase K to reverse the formaldehyde crosslinking. And the precipitated RNA was finally extracted and examined by qRT-PCR.

### Western blot

Osteosarcoma MG63 and U2OS Cells were lysed with M-PER Protein Extraction Reagent (Pierce) supplemented with protease inhibitor cocktail (Calbiochem). Protein concentrations were measured with BCA assay (Pierce) and equalized with the extraction buffer. Equal amount of the extracts were loaded and subjected to SDS-PAGE, transferred onto nitrocellulose membranes, and then blotted. Antibodies specific to human Bcl-2 and β-actin, and horseradish peroxidase-coupled secondary antibodies were reported previously [33].

### Statistical analysis

Data are shown as mean ± s.d. Student’s *t*-test was used to analyze statistical comparisons between groups, and a two-tailed *p*<0.05 was considered to be statistical significant. Spearman's rank correlation coefficient assay in SPSS 17.0 was used to analyze the correlation between circUBAP2 expression and clinical osteosarcoma stages. Kaplan-Meier survival analysis with log-rank test in SPSS 17.0 was used to analyze the overall survival in osteosarcoma patients with the median value of circUBAP2 expression as the cutoff. Pearson’s correlation coefficient assay in SPSS 17.0 was used to analyze the correlation between circUBAP2 expression and miR-143 expression in osteosarcoma tissues.
